# Morin hydrate reduces survival and fertility, delays development and weakens lipid reserves in *Aedes aegypti*

**DOI:** 10.1111/mve.12805

**Published:** 2025-04-19

**Authors:** Luan Valim dos Santos, Elaine Rodrigues Miranda Nery da Silva, Matheus Silva Caiado, Renan Albuquerque Camasmie, Herbert Marcusi Souza de Agustini, Raquel do Nascimento de Souza, Bruno Guimarães Marinho, Rosane Nora Castro, Mario Geraldo de Carvalho, Emerson Guedes Pontes

**Affiliations:** ^1^ Departamento de Bioquímica, Instituto de Química Universidade Federal Rural do Rio de Janeiro Seropédica Brazil; ^2^ Departamento de Ciências Fisiológicas, Instituto de Biologia Universidade Federal Rural do Rio de Janeiro Seropédica Brazil; ^3^ Departamento de Química Orgânica, Instituto de Química Universidade Federal Rural do Rio de Janeiro Seropédica Brazil

**Keywords:** flavonoid, larvae, lipids, mosquito, triacylglycerol

## Abstract

The *Aedes aegypti* mosquito is generally associated with arboviruses that cause yellow fever, dengue, zika and chikungunya. The most efficient way to control their populations is through application in breeding sites of highly toxic insecticides that can also impact non‐target organisms and generate resistant populations. Therefore, the use of compounds is desirable. Morin hydrate has broad pharmacological applications based on its antioxidant potential, in addition to not having negative effects on mammals. Therefore, the objective of the present study was to investigate the effects of morin hydrate on *A. aegypti* survival, pupation rate, egg laying, triacylglycerol reserves and expression of proteins related to lipid metabolism 24 h after exposure of larvae. For this, rearing media containing *A. aegypti* larvae with different concentrations of morin hydrate were formulated to evaluate the lethal concentration. Calculation of the expected lethal concentrations showed LC_25_ of 52.692 μM, LC_40_ of 111.121 μM, LC_50_ of 174.775 μM, LC_75_ of 575.083 μM and LC_90_ of 1685.936 μM. Twenty‐four hours after treatment with morin hydrate, surviving larvae were transferred to morin‐free water with food, and their pupation rate and fertility were evaluated. We observed that an increase in the concentration of morin hydrate induced a dose‐dependent reduction in survival, doubled pupation time in survivors and reduced the number of eggs laid by treated females during the larval stage by approximately 30% at concentrations exceeding 100 μM. From this, the impact of 24 h on the triacylglycerol (TAG) stock was evaluated, in addition to evaluating the expression of proteins involved in lipid metabolism. Larvae 24 h after treatment with 100 μM morin showed a reduction in TAG reserves of approximately 17%, while at 175 μM, there was a reduction of more than 33% in stocks, and at 500 μM there was a reduction of 61%. Furthermore, the lipolytic proteins TAGL1 and HSL were upregulated, while the lipogenic proteins FAS1, DGAT1 and GPAT1 were downregulated. Insulin‐like receptors were also downregulated, unlike AKHr, which was also upregulated. These data demonstrate that morin hydrate reduces the survival and fertility of *A. aegypti* by affecting its lipid metabolism. Morin hydrate did not exhibit toxicity toward non‐target organisms, demonstrating interesting potential for the control of mosquito populations.

## Graphical abstract

- Morin hydrate promoted mortality and reduced the rate of pupation and egg laying in *Aedes aegypti* larvae that survive treatment.
- Morin hydrate reduces the levels of triacylglycerol, the main lipid reserve in *A. aegypti* larvae.
- The expression of proteins involved in lipogenesis and lipolysis was downregulated and upregulated, respectively, in larvae that survived treatment.

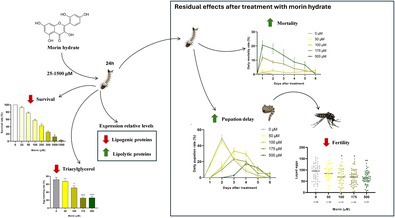

## INTRODUCTION

Arboviruses are important causes of disease in humans worldwide, mostly in tropical and subtropical regions where epidemics caused by arboviruses occur in persistent urban endemic cycles, generating economic and public health impacts (Soni et al., 2023 Jul 11). The *Aedes aegypti* mosquito is generally associated with arboviruses that cause yellow fever, dengue, zika and chikungunya, and its anthropophilic habit and vector competence increase the potential for the occurrence of these diseases (Weaver & Reisen, 2010). It is estimated that between 1975 and 2022, expenses resulting from the treatment of diseases transmitted by *A. aegypti* and their sequelae reached US$ 310.8 billion worldwide (Roiz et al., 2024). There is a consensus in the literature that control of the mosquito vector is the best prevention tool, given the absence of vaccines or viable treatments for most of these arboviruses (Soni et al., 2023).

Lipids are essential for maintaining the regular functions of insect metabolism, since they are involved in embryogenesis, growth, development, metamorphosis, diapause, reproduction, prolonged flight and survival during winter and periods of food shortages (Arrese & Soulages, 2010; Gondim et al., 2018). Among the most abundant lipids, those from the glycerolipid class stand out as the most abundant lipid family in insects, with numerous cellular functions. However, their function as energy stores stands out (Kaczmarek et al., 2024). Fat bodies contain abundant reserves of triacylglycerol (TAG), which is highly efficient in conserving energy during periods of nutritional abundance (Arrese & Soulages, 2010). In this context, the mobilization of these reserves is efficiently modulated through hormonal actions (Toprak, 2020), and activation of lipases is fundamental, since they act in the complete degradation of TAG, releasing glycerol and fatty acids, ensuring energy homeostasis (Cerk et al., 2017; Pirahanchi & Sharma, 2019). Given the importance of lipids for insects, damage to this system can make mosquito survival impossible.

The most efficient way to control *A. aegypti* populations generally involves applying organophosphate‐based insecticides. However, their high toxicity impacts non‐target organisms and generates selective pressures for the development of resistant populations (Arias‐Castro et al., 2020). Therefore, strategies that use natural products are recommended to reduce the impact on vertebrate wildlife and humans (dos Santos et al., 2023). The use of natural compounds is an important alternative to conventional products and also in combination with them (Pavela et al., 2019). Morin hydrate (3,5,7,2′,4′‐pentahydroxyflavone) is a polyphenol from the flavonol class that is isolated from several plant families, such as the Moraceae family. It has broad pharmacological applications related to its antioxidant potential (Rajput et al., 2021). Furthermore, the use of nanoparticles containing morin did not compromise the viability of hepatocytes in vitro, in addition to being efficient in the treatment of diabetic mice by restoring liver, kidney and pancreatic functions, reducing tissue vacuolization and increasing cellular glucose uptake (Hua et al., 2024). Additionally, it has been observed to impact lipid metabolism by inhibiting pancreatic lipases and lipid synthesis (Li & Tian, 2004; Venkateish et al., 2024).

No studies have demonstrated the effects of morin hydrate in in vivo insect models; however, there are indications of its inhibitory potential on insect enzymes. It has been reported that the activity of ecdysone 20‐monooxygenase, a key enzyme in the molting process, is inhibited in a dose‐dependent manner in homogenates of *A. aegypti*, *Drosophila melanogaster and Manduca sexta* (Mitchell et al., 1993). Similarly, the activity of mitochondrial transhydrogenase was assessed using mitochondria isolated from the midgut of *M. sexta* larvae, and dose‐dependent inhibition was reported in the presence of morin (Vandock et al., 2012).

As the compound is already being used as an alternative treatment for some human dysfunctions, its use as an alternative insecticide would not be a harsh threat for vertebrates. Therefore, the objective of the present study was to investigate the effects of morin hydrate (3,5,7,2′,4′‐pentahydroxyflavone) on the survival of *A. aegypti* larvae, pupation rate and adult fertility following treatment, as well as to evaluate lipid reserves and the expression of proteins involved in synthesis and hydrolysis pathways.

## METHODS

### Rearing of mosquitoes

All experiments were conducted using larvae cultivated from a colony of *A. aegypti* (Rockefeller strain) maintained at the Laboratory of Biochemistry and Molecular Biology of Arthropods (LBBMA), Department of Biochemistry, Institute of Chemistry of Federal Rural University of Rio de Janeiro, Brazil. The colony was kept under a 12:12 h photoperiod at a constant temperature of 27 ± 1°C and relative humidity of 80 ± 10%. The larvae were fed mashed cat food (Whiskas), and adults were provided with sugar solution ad libitum. For egg laying, a blood meal was offered to the mosquitoes 4 days post‐emergence of adults. All experiments were performed in accordance with animal experimentation standards and ethics and were approved by the Ethics Committee of Animal Use (CEUA) of Universidade Federal Rural do Rio de Janeiro under protocol number 006/2022.

### Morin hydrate treatment

Approximately 200 larvae were reared in 1.5 L of dechlorinated water until they reached the L3 stage, with cat food provided ad libitum. The larvae were then transferred to containers holding dechlorinated water and commercial morin hydrate (Merck, purity <85%) at concentrations ranging from 10 to 2500 μM. Morin hydrate was initially dissolved in 0.1% absolute ethanol and subsequently diluted in water. A density of one larva per mL of the solution was used. A control group of only 0.1% absolute ethanol (0 μM) was established. Larval density was controlled throughout all experiments, maintaining one larva per milliliter of rearing medium. Larvae were kept under fasting conditions and exposed to morin hydrate for 24 h.

### Mortality and pupation assays

The survival of larvae subjected to treatment with different concentrations of morin hydrate was assessed after 24 h in four independent replicates, each consisting of four containers with 10 larvae in 10 mL of rearing medium containing varying concentrations of morin hydrate. For the daily mortality and pupation assays, larvae were treated with 50, 100, 175 and 500 μM of morin hydrate for 24 h. The concentrations used were based on the calculated lethal concentrations: LC_25_ at 52.692 μM, LC_40_ at 111.121 μM, LC_50_ at 174.775 μM, LC_75_ at 575.083 μM. After this period, the surviving larvae were transferred to morin‐free water, and 10 mg of food (1 mg per larva) was provided. Counting was performed daily at regular 24‐h intervals for 6 days.

### Egg counting assay

For the egg counting assay, larvae treated with morin hydrate for 24 h were transferred to rearing medium with clean water. Pupae that emerged on days 3 and 4 after treatment were transferred to cages. Adults emerging from these pupae were fed ad libitum with 10% sucrose solution. Four‐day‐old adults were given a blood meal, and after 24 h, engorged females were transferred to oviposition containers lined with moist cotton and filter paper to count the number of eggs laid by each female at 72 h post‐blood meal.

### Determination of triacylglycerol and total protein

For the determination of triacylglycerol (TAG) content, 10 larvae treated with different concentrations of morin hydrate were homogenized in 200 μL of distilled water in five independent replicates. The TAG content was measured using a commercial triglyceride monoreagent kit (Bioclin) following the manufacturer's protocol, where 10 μL of larval homogenate was mixed with 200 μL of the colour reagent and incubated at 37°C for 30 min. The absorbance was measured at 540 nm, and the amount of TAG was calculated by comparing the absorbance to a standard TAG curve.

The results were normalized to the total protein content of the samples, which was measured using the modified Lowry method (Lowry et al., 1951). Samples (2 μL) were added to 200 μL of Lowry solution and 20 μL of Folin–Ciocalteu reagent (Sigma‐Aldrich). Absorbance was measured at 660 nm, and protein content was calculated by comparing the absorbance to a standard curve of bovine serum albumin (Sigma‐Aldrich).

### RNA extraction and cDNA synthesis

After 24 h of treatment with 50, 100, 175 or 500 μM of morin hydrate, 10 larvae were homogenized in 1 mL of TRIzol Reagent (Invitrogen) in quadruplicates. Total RNA extraction was performed according to the manufacturer's protocol and in accordance with the MIQE guidelines (Bustin et al., 2009). RNA was quantified using a NanoDrop ND‐2000 spectrophotometer (Thermo Fisher Scientific). All samples had A260/A280 and A260/A230 ratios close to 2.0 ± 0.1, and RNA integrity was analysed via agarose gel electrophoresis. For cDNA synthesis, 1 μg of RNA was treated with 1 U of DNase I (Sigma‐Aldrich) for 30 min at 37°C, and the reaction was stopped by adding 50 mM EDTA followed by incubation at 70°C for 10 min. A High‐Capacity cDNA Reverse Transcription Kit (Thermo Fisher Scientific) was used for cDNA synthesis according to the manufacturer's protocol.

### Quantitative PCR (qPCR)

The qPCR was performed with a StepOnePlus system (Thermo Fischer Scientific) using Power SYBR™ Green Master Mix (Applied Biosystems). Primers of triacylglycerol lipase‐1 (TAGL1), Brummer lipase (Bmm), hormone‐sensitive lipase (HSL), diacylglycerol acyltransferase 1 (DGAT1), glycerol‐3‐phosphate acyltransferase‐1 (GPAT1), adipokinetic hormone receptor (AKHr) and insulin‐like receptor (ILr) were used as described by Dou et al. (2023). The fatty acid synthase‐1 (FAS1) primer was used as described by Alabaster et al. (2011). Additional information about the primers is described in Table S1, including identification in VectorBase, oligo sequence, amplification efficiency, cDNA curve slope and *r* ^2^. The reactions were performed in a MicroAmp Fast Optical 96‐Well Reaction Plate with Barcode (Applied Biosystems), using the equipment's default configuration: 10 min at 95°C, followed by 40 cycles of 15 s at 95°C and 1 min at 60°C, and a dissociation curve. A negative control was performed to determine whether the reaction mixtures were contaminated with exogenous DNA. Actin and α‐Tubulin were used as reference genes in all samples tested. The relative expression of the genes and the expression were calculated based on both the reference genes and the control sample subjected to 0 μM morin.

### Rearing of mice and acute toxicological evaluation of morin hydrate

Male (*n* = 13) *Swiss* mice were obtained from the Central Biotherium of Universidade Federal Rural do Rio de Janeiro. All experiments were performed in accordance with animal experimentation standards and ethics and were approved by the Ethics Committee of Animal Use (CEUA) of Universidade Federal Rural do Rio de Janeiro under protocol number 006/2022. The animals were maintained under standard conditions (ventilated room, 22 ± 3°C, 12 h light–dark cycle), with free access to food and water.

Acute toxicological evaluation was carried out according to the guideline developed by the Organization for Economic Cooperation and Development (OCDE, 2022). Male *swiss* mice (*n* = 13 animals) fasted for 12 h. After this period, eight males were orally exposed to 300 mg/kg of morin hydrate (solubilized in 0.9% saline solution) and monitored for symptoms of toxicity and mortality at 30 min, 1, 2, 3, 4, 5, 6, 12 and 24 h. The control group (*n* = 5) was orally exposed to 0.9% saline solution.

### Data analysis

Graphical representations and statistical analyses were performed using the Prism 8.0.2 software (GraphPad Software). All results are presented as the mean ± standard error of the mean (SEM). Probit analysis was conducted using Finney's calculator (Mekapogu, 2021). Analysis of variance (ANOVA) was performed to compare the control group with the morin‐treated group, with the statistical significance set at *p* < 0.05.

## RESULTS AND DISCUSSION

### Effects of morin hydrate on the survival and fitness of mosquitoes

**FIGURE 1:**
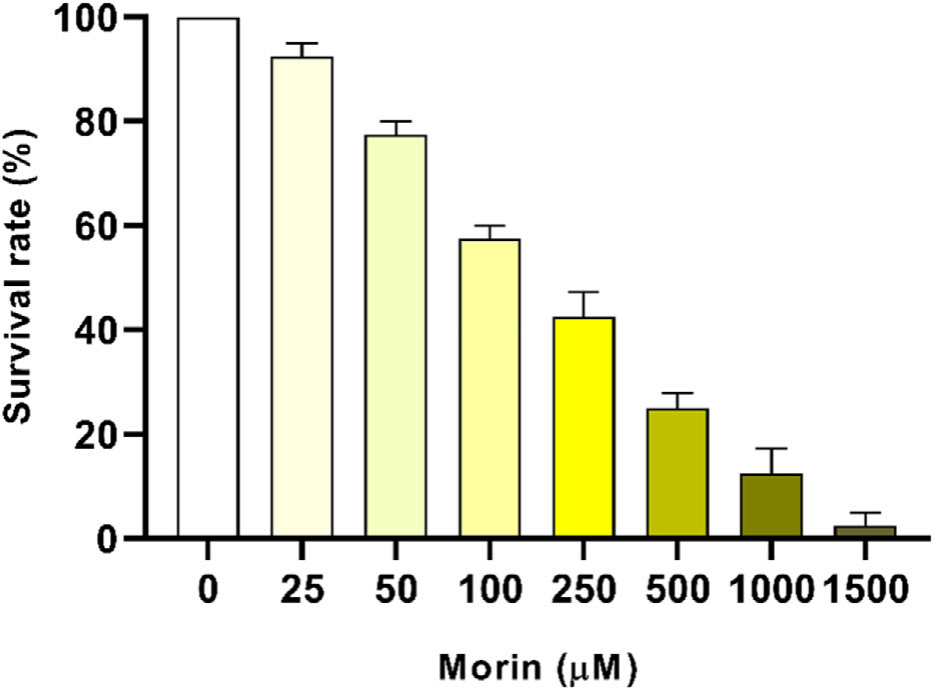
Survival rate of larvae treated with different concentrations of morin hydrate. L3 stage larvae were reared in solutions with different concentrations of morin, and the survival rate after 24 h was determined. Values are reported as the percentage of mean survival of four independent biological replicates ± SEM (*n* = 16).

There is few information on the effects of morin on insects, especially mosquitoes. Therefore, we decided to observe the effects of different morin hydrate concentrations on yellow fever mosquito larvae. In the 24‐h exposure tests, we observed a gradual reduction in larval survival depending on morin concentration (Figure 1). Furthermore, a reduction in larval survival was observed only at concentrations above 25 μM, while mortality of all larvae occurred at concentrations greater than 1800 μM after this period. The calculation of the expected lethal concentrations showed LC_25_ of 52.692 μM, LC_40_ of 111.121 μM, LC_50_ of 174.775 μM, LC_75_ of 575.083 μM and LC_90_ of 1685.936 μM. The observed toxicity might have been related to the high reactivity of flavonoids (Pavela et al., 2019) and their inhibitory potential against detoxification enzymes in insects (Azuma, 2014; Muthu et al., 2015). In the literature, the use of plant extracts to evaluate insecticidal activity is commonly observed, and in most cases, this potential has been associated with the presence of flavonoids. However, the larvicidal action of flavonoids isolated from plants has been reported in the literature, such as pectinaringenin (5,7‐dihydroxy‐6,4′‐dimethoxyflavone) against *Anopheles stephensi* and *Earias vittella* (Muthu et al., 2015), caused by the inhibition of detoxification enzymes such as glutathione S‐transferase and esterases (Azuma, 2014). Another case involving enzyme inhibition was reported for *Spodoptera frugiperda*, where inhibition of acetylcholinesterase was observed by the action of quercetin, an isomer of morin (3,5,7,3′,4′‐pentahydroxyflavone) (Herrera‐Mayorga et al., 2022). It was also reported that morin and quercetin supplementation with the addition of ethylparaoxon and cypermethrin reduced weight gain and survival of *Helicoverpa armigera* larvae (Aurade et al., 2011). In other words, like morin, other flavonoids have also been reported as having larvicidal potential.

**FIGURE 2:**
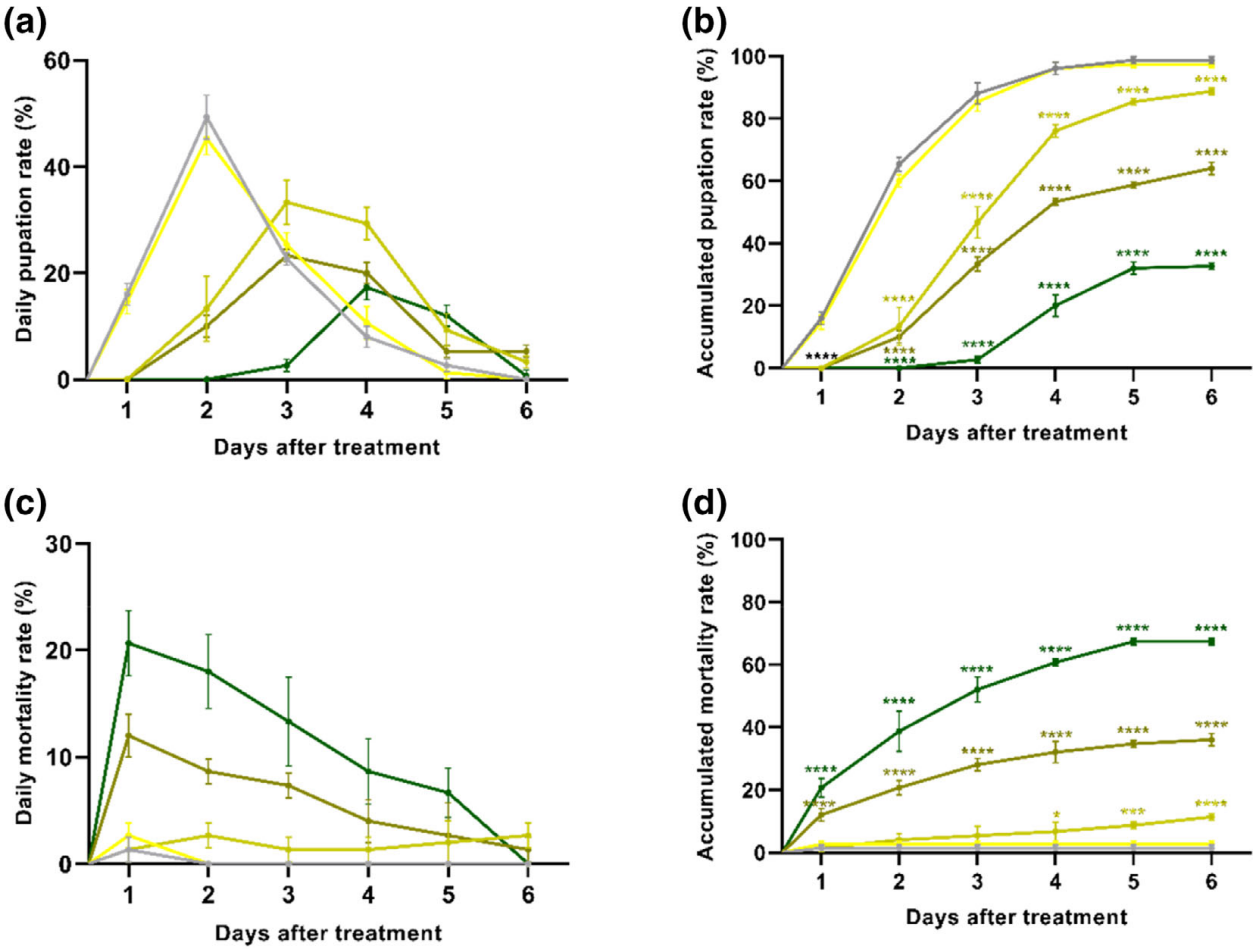
Daily pupation and mortality rates following morin hydrate treatment. After treatment for 24 h, the surviving larvae were washed and transferred to a medium containing clean water. Daily pupation (a), accumulated pupation (b), daily mortality (c) and accumulated mortality (d) rates were noted at 24‐h intervals. Statistical differences relative to the control (0 μM morin hydrate) were determined using two‐way ANOVA. Values are reported as the percentage of pupation and mean mortality of three independent biological replicates ± SEM (*n* = 12). Bars annotated with asterisks are significantly different from the control (0 μM) at ***p < 0.001 in b and d.

To evaluate the efficiency of the treatment and the impacts on the developmental phases, the effect on these larvae was observed after a 24‐h treatment with morin hydrate. The treated larvae were transferred to medium containing clean water and food. We chose to use concentrations close to LC_25_, LC_40_, LC_50_ and LC_75_. Using a value close to LC_90_ proved unfeasible because it generated very high mortality and restricted the amount of material. Pupation and mortality rates were determined in days after treatment with different concentrations of morin (Figure 2), where on the sixth day, all larvae in the treatment group had turned into pupae or died.

Our results showed that almost half of the larvae in the control group and the 50 μM group became pupae on the second day after treatment, with rates of 49.3% and 45.3%, respectively (Figure 2a). In contrast, the peak pupation rate of larvae treated with 100 and 175 μM occurred only on the third day, whereas larvae treated with 500 μM had peak pupation only on the fourth day (Figure 2a). In addition to the delay in pupation time, there was a significant reduction in the accumulation rates observed with higher concentrations of morin. After 6 days, almost all larvae became pupae at concentrations of 0 and 50 μM, showing a small reduction in the total number of pupae observed at 100 μM, which intensified with the increase in the concentration of morin hydrate, with accumulation rates of approximately 64% and 33% at 175 and 500 μM, respectively (Figure 2b). These results suggest an imbalance in animal homeostasis and that important metabolic pathways may have been affected. Data in the literature indicate that flavonoids can impact the development of *A. aegypti* by reducing the activity of glutathione S‐transferase Noppera‐bo (Inaba et al., 2022) and ecdysone 20‐monooxygenase dependent on cytochrome P‐450 (Mitchell et al., 1993), which are involved in the synthesis of ecdysone precursors and activators, respectively. Ecdysone is a key regulator of developmental timing, larval growth and metamorphosis in holometabolic insects (Kannangara et al., 2021). Therefore, the increase in the concentration of morin hydrate may have been related to the reduction in ecdysone synthesis due to enzymatic inhibition, thus delaying the pupation rate reported in our study.

**FIGURE 3:**
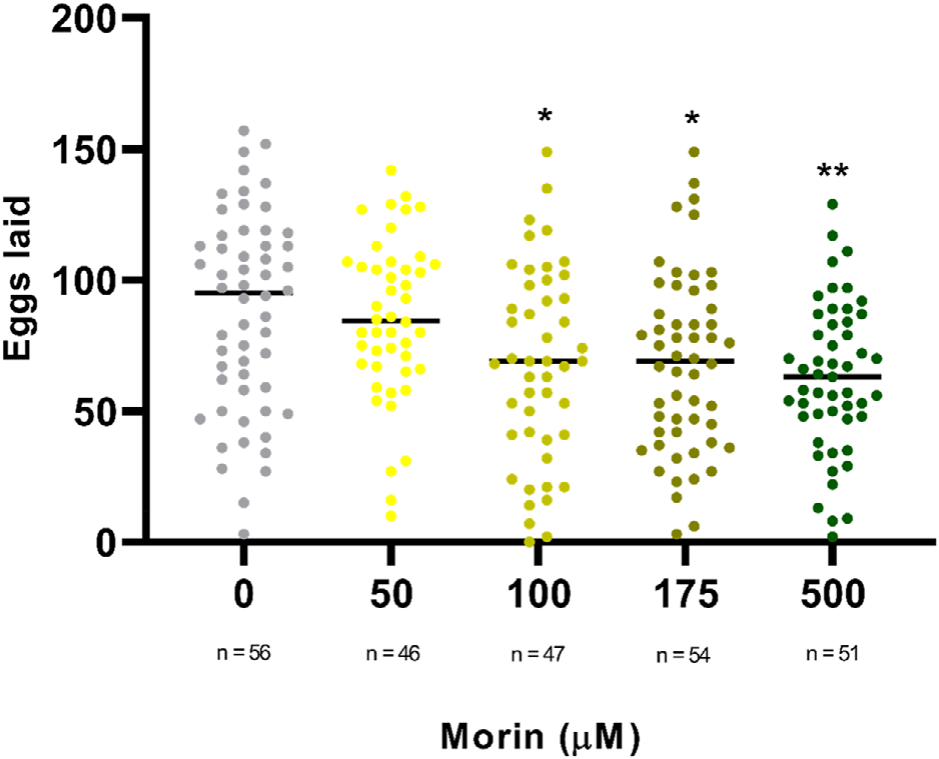
Egg laying by females subjected to different concentrations of morin hydrate during the larval stage. L3 stage larvae of *Aedes aegypti* were subjected to 24‐h treatment with different concentrations of morin hydrate. The surviving larvae after treatment that reached the adult stage were fed with blood, and the number of eggs laid was counted. Values represent the median of four independent biological replicates ± SEM. Statistical differences were determined using the Kruskal‐Wallis test (*p < 0.05, **p < 0.01, ***p < 0.001 and ****p < 0.0001).

**FIGURE 4:**
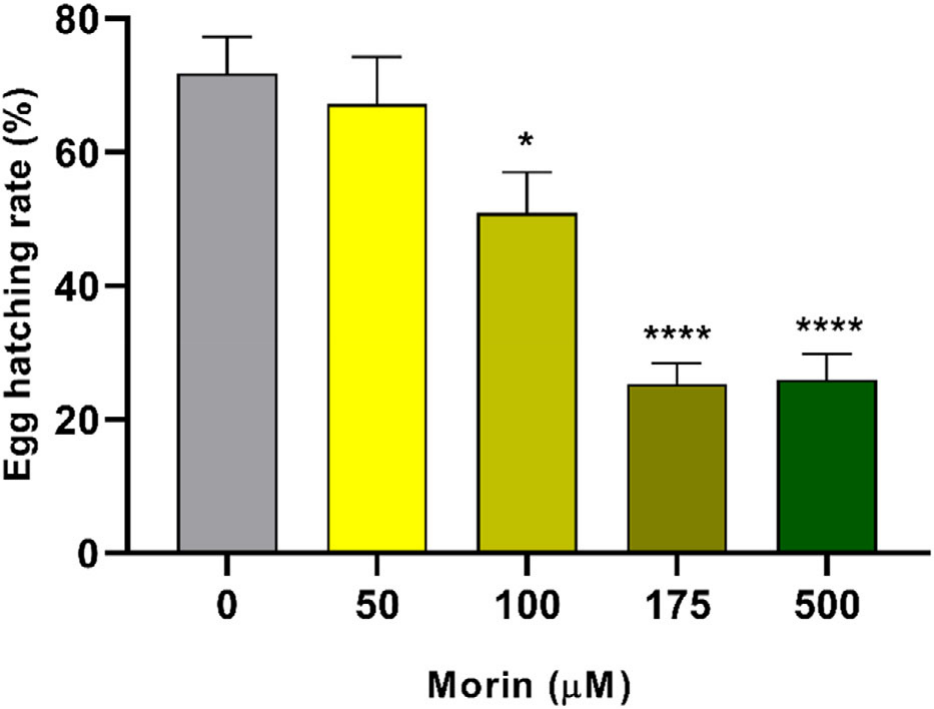
Hatching rate of eggs laid by females treated with different concentrations of morin. L3 stage larvae of *Aedes aegypti* were subjected to 24‐h treatment with different concentrations of morin hydrate, and the larvae that reached the adult stage were fed with blood after 96 h. Eggs were collected, and the number of larvae hatched from these eggs were used to calculate the percentage. Values represent the mean of three independent biological replicates ± SEM (*n* = 15). Statistical differences were determined by one‐way ANOVA (*p < 0.05, ***p < 0.001 and ****p < 0.0001).

We determined whether morin hydrate treatment was able to affect the reproductive capacity of *A. aegypti*. For this purpose, adults originated from larvae that survived different concentrations of morin hydrate were separated. On the fourth day after emerging as adults, they were fed with blood and the number of eggs was determined after 72 h. The data in Figure 3 show that females treated with 100 μM and 175 μM of morin hydrate laid approximately 28% fewer eggs in comparison with the control group (*p* < 0.05), while females treated with 500 μM laid 35% fewer eggs (*p* < 0.01). After counting, the hatching rate was calculated (Figure 4), where a reduction of more than 20% in the hatching rate of eggs laid by females that were treated with 100 μM during their larval stages was observed in relation to the control, while eggs from females that were treated with 175 μM and 500 μM showed a reduction in hatching rate of more than 46%.

These data show that treatment with morin hydrate causes larval mortality in a dose‐dependent manner and that even after treatment, the larvae that survive were impacted both in development and in the generation of offspring, even without being exposed for more than 24 h. In other words, the administration of morin had a long‐term and systemic residual effect on the treated larvae.

### *Morin hydrate impacts the lipid metabolism of* Aedes aegypti

**FIGURE 5:**
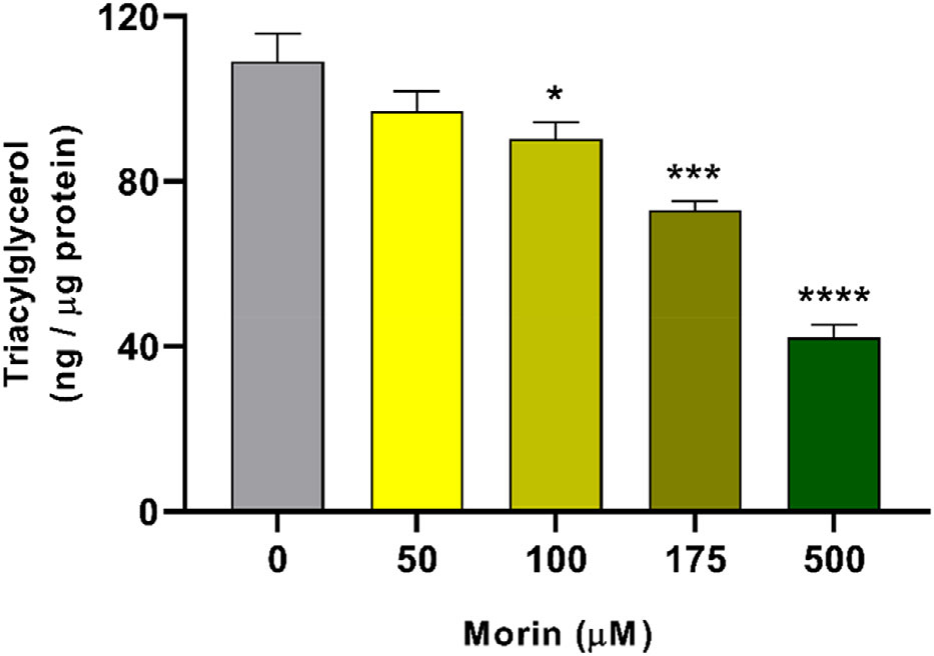
Triacylglycerol content in whole *Aedes aegypti* larvae after 24 h of exposure to different morin hydrate concentrations. L3 larvae were subjected to 24‐h treatment with different concentrations of morin hydrate, and the total TAG of the entire larva was quantified and normalised to total proteins. Values represent the mean of four independent biological replicates ± SEM (*n* = 12). Statistical differences were determined using one‐way ANOVA (*p < 0.05, ***p < 0.001 and ****p < 0.0001).

Deficits in lipid metabolism have a strong negative impact on the fertility and survival of insects, of which TAG stocks are fundamental for the regulation and maintenance of homeostasis (Alabaster et al., 2011; Arrese & Soulages, 2010; Gondim et al., 2018). Therefore, investigating how these lipid stores are maintained in the face of the challenge with morin hydrate provides an opportunity to understand its effects on energy metabolism. We observed that larvae reared with 50 μM showed no difference in relation to the control, unlike higher concentrations (Figure 5). Larvae reared with 100 μM suffered a reduction of around 17%, while at 175 μM there was a reduction of more than 33% of stocks and at 500 μM the decrease was 61% (Figure 5). These data demonstrate that TAG mobilization occurs in a dose‐dependent manner of morin hydrate and that the stored energy is directed to deal with the stress generated.

Variations in TAG content caused by stress have already been reported in the literature. The stress caused by a high larval density in *A. aegypti* generates an increase in the occurrence of TAG in pupae and adults, in addition to generating an increase in the body size of adult mosquitoes (Silva et al., 2021). On the other hand, the suppression of an energetic diet during the adult life of yellow fever mosquitoes generated a large reduction in TAG stocks after 3 days and led to mortality after 7 days (dos Santos et al., 2024). The use of *Cymbopogon citratus* essential oil caused mortality of the beetle *Callosobruchus maculatus*, in addition to reducing the levels of TAG and total fatty acids by around 60%, thus harming the fertility of treated females (Alves et al., 2015; Alves et al., 2019). Additionally, it is known that lipids are mobilized during immunological depression as an energy source for the biogenesis of membranes at sites of infections and hemocytes, supporting other immunological functions in fat bodies (Arrese & Soulages, 2010; Wrońska et al., 2023).

**FIGURE 6:**
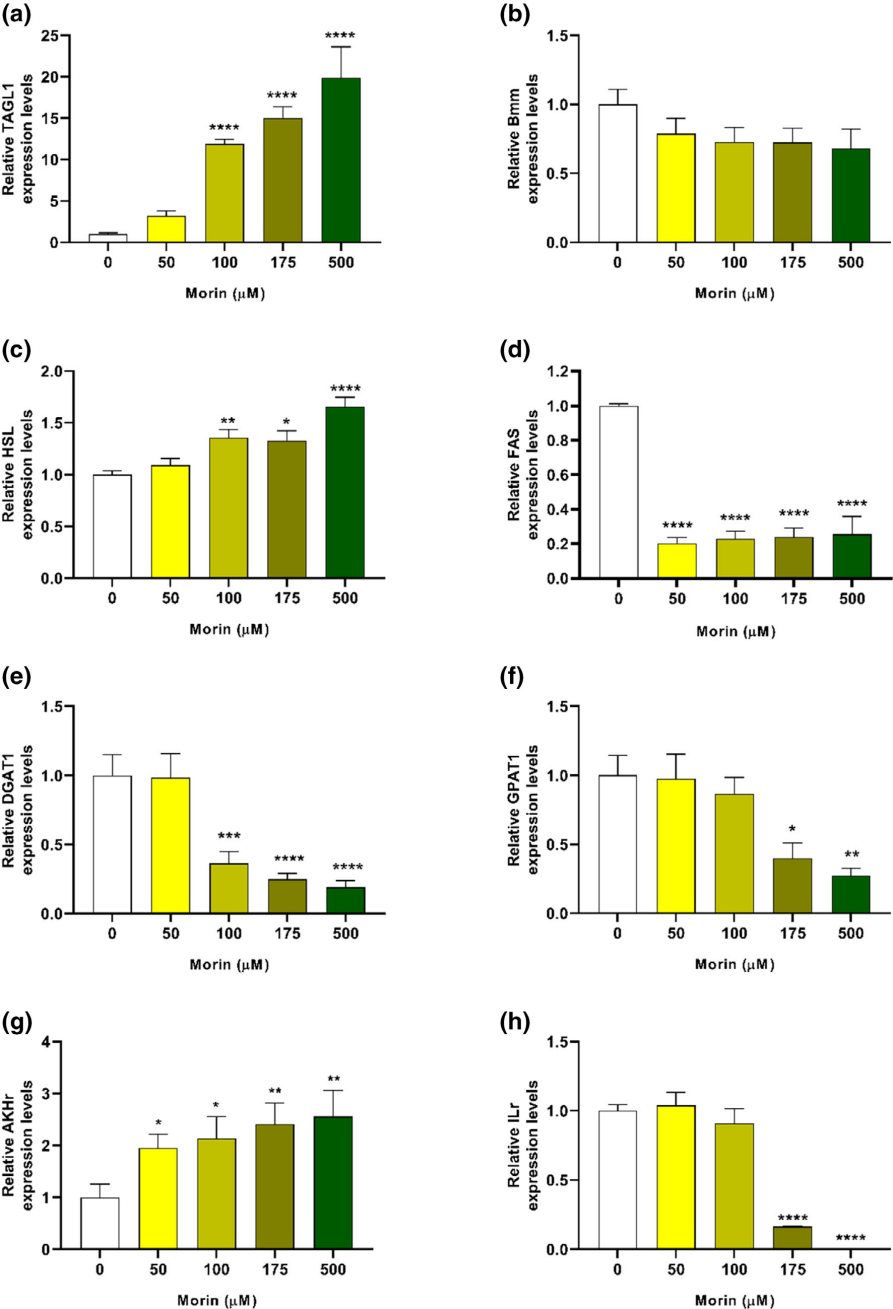
Expression analysis of genes in *A. aegypti* after 24 h of exposure to different concentrations of morin hydrate. The expression levels of triacylglycerol lipase 1 (a), brummer lipase (b), hormone‐sensitive lipase (c), fatty acid synthase (d), diacylglycerol acyltransferase 1 (e), glycerol‐3‐phosphate acyltransferase (f), adipokinetic hormone receptor (g) and insulin‐like receptor (h) were relative to the values of larvae at 0 μM (1.0). Expression levels were normalized to those of the reference genes, actin1 and α‐tubulin. Values are reported as the percentage of peak expression and represent the mean of four independent biological replicates ± SEM (*n* = 8). Statistical differences relative to the control (0 μM) were determined using one‐way ANOVA (*p < 0.05, **p < 0.01, ***p < 0.001 and ****p < 0.0001).

To understand the contribution of the enzymes involved, the expression levels of different enzymes that are involved in lipid metabolism (Figure 6) were evaluated in larvae treated with morin hydrate for 24 h. We selected proteins with lipolytic action, among them: brummer lipase (Bmm) and triacylglycerol lipase 1 (TAGL1), which are involved in the hydrolysis of TAG [30,31]; hormone‐sensitive lipase (HSL), which hydrolyses diacylglycerol (DAG) after hormonal stimulation (Grönke et al., 2005; Heier & Kühnlein, 2018); and hormone‐sensitive lipase (HSL), which hydrolyses DAG after hormonal stimulation (Gondim et al., 2018; Heier & Kühnlein, 2018). We also selected three proteins involved in lipid synthesis: fatty acid synthase (FAS1), which synthesizes fatty acids (Alabaster et al., 2011); diacylglycerol acyltransferase 1 (DGAT1), which converts DAG into TAG through esterification with a fatty acid (Heier & Kühnlein, 2018); and glycerol‐3‐phosphate acyltransferase 1 (GPAT1), which catalyses the first step of TAG synthesis and also acts as a rate‐limiting enzyme for the de novo pathway of glycerophospholipid synthesis (Yamashita et al., 2014). The expression of the adipokinetic hormone receptor (AKHr) and insulin‐like receptor (ILr) was also observed, which involves important hormones in multiple signalling during insect development (Alves‐Bezerra et al., 2016; Arrese et al., 1996; Gulia‐Nuss et al., 2011; Lu et al., 2018; Patel et al., 2006; Perez‐Hedo et al., 2014; Roy et al., 2007).

Triacylglycerol lipase 1 (TAGL1) expression did not change at 50 μM in relation to the control. However, at concentrations above 100 μM, highly significant increases in expression were observed (Figure 6a) in relation to the control, with an increase of approximately 11 times at 175 μM and an increase of more than 15 times at 500 μM. These data imply greater mobilization of TAG reserves, which corroborates the consumption observed in Figure 5. On the other hand, bmm also hydrolysed TAG, but it did not show significant variation in any of the treatments (Figure 6b). TAGL1 is regulated in response to rapid changes in lipid demands, whereas bmm is related to prolonged fasting (Grönke et al., 2005; Grönke et al., 2007), which has already been reported in adult *A. aegypti* mosquitoes (dos Santos et al., 2024). Therefore, it makes sense to conclude that TAGL1 is associated with homeostasis, which in turn regulates TAG stores, while bmm is restricted to diet‐related conditions during the larval stage. Notably, during adulthood, bmm can be modulated by post‐vitellogenic factors (dos Santos et al., 2024; Dou et al., 2023). HSL did not show any variation at 50 μM in relation to the control, but at 100 and 175 μM, there were increases of approximately 34% and of more than 65% at 500 μM (Figure 6c).

There was a reduction of over 75% in the expression of FAS1 at all concentrations tested (Figure 6d). A curious finding was that FAS1 was highly sensitive to the treatment because, at the concentration of 50 μM, the behaviour was similar to that of the higher concentrations, although this concentration was not lethal and did not negatively impact the fitness of *A. aegypti*. Fatty acids are considered fundamental to the physiology of insects, which use them mainly as an energy source for movement and for immune system activity (Kaczmarek et al., 2024). Nonetheless, it has been reported that morin is highly efficient in inhibiting animal FAS in vitro (Li & Tian, 2004). Therefore, we believe the reduction in lipid stocks is due to the inability of larvae to synthesize fatty acids and that to meet the demands, it is necessary to consume TAG stocks to release the fatty acids as an energy source from breaking ester bonds (Heier & Kühnlein, 2018). The reduction in FAS1 expression at all concentrations reflects the high catabolic metabolism, which consumes energy stores and inhibits the synthesis of fatty acids.

Other lipid synthesis pathways were affected by morin hydrate treatment, reinforcing the dose‐dependent catabolic activity. DGAT1 expression levels were significantly lower at concentrations above 100 μM, with reductions between 65 and 80% (Figure 6e). In other words, the reduction in TAG stocks was also compromised by the reduction in its synthesis. GPAT1, which is involved in the de novo synthesis of glycerolipids by catalysing the rate‐limiting reaction in the conversion of glycerol‐3‐phosphate and long‐chain acyl‐CoA into lysophosphatidic acid (Yu et al., 2018), also showed reduced expression at concentrations above 175 μM (Figure 6f). There was a direct relationship between GPAT expression levels and TAG reserves. Gene silencing of this protein in *Rhodnius prolixus* reduced the levels of these lipid reserves in the intestine and fat body (Alves‐Bezerra et al., 2017). Therefore, our results indicate that lipid synthesis pathways were downregulated by morin hydrate treatment.

The contribution of signalling pathways to the synthesis and mobilization of lipid metabolism was also evaluated. The expression of receptors of hormones AKH and IL was observed in *A. aegypti* larvae treated with morin hydrate. Normally, energy balance is regulated through the opposition between insulin‐like peptides (ILP), which in general promote nutrient storage, and AKH, which is related to the mobilization of carbohydrates and lipids (Alves‐Bezerra et al., 2016; Gulia‐Nuss et al., 2011; Lu et al., 2018; Sharma et al., 2019). We observed that AKHr expression showed a progressive increase as the treatment concentration increased, doubling the expression observed at 50 and 100 μM, increasing it by 140% and 155% at 175 and 500 μM, respectively (Figure 6g). ILr caused very abrupt reductions in concentrations above LD_50_. The expression level in larvae treated with 175 μM corresponded to only 16% of the expression level of the control group, while at 500 μM the level was only 0.1% of the control group (Figure 6h). The increase in AKHr may be related to the increase in lipase activities, since AKH, which is normally related to mammalian glucagon, activates complex signalling pathways that trigger the activation of lipases such as TAGL1, bmm and HSL (Arrese et al., 1996; Toprak, 2020), as well as inhibiting lipogenic enzymes such as DGAT1 and GPAT1 (Alves‐Bezerra et al., 2016; Toprak, 2020). The role of ILPs is very complex. They are key elements of insect growth, reproduction, regulation of stress responses and lifespan, in addition to regulating levels of metabolites circulating in the hemolymph (Wu & Brown, 2006). With regard to metabolism, ILr activation stimulates the AKT pathway, which inhibits lipolytic pathways and improves lipogenic pathways (Roy et al., 2007; Sharma et al., 2019). ILr knockout in *Tribolium castaneum* larvae generated high mortality, while induction during the pupal stage caused inhibition in the number of eggs laid (Sang et al., 2016), while in *A. aegypti* adults it generated a reduction in energy reserves for eggs and, consequently, the viability of offspring (Gulia‐Nuss et al., 2011). Therefore, the expression levels of the receptors observed in our experiments confirm the impairment of metabolism due to the action of morin hydrate.

The reductions of TAG stocks are directly related to mortality and reduced fertility. However, the specific mechanisms that generated the mobilization are still unknown. More studies are needed to understand the mechanisms of action that generated these effects in *A. aegypti*.

### Toxicological evaluation of morin hydrate

To assess the effects of morin hydrate in vertebrates, we conducted toxicological assays in mice using a dose approximately 1000 times higher than that used in mosquito assays. No symptoms of intoxication, such as seizures, hyperactivity, loss of righting reflex, altered respiratory rate or sedation, and no deaths were observed. This outcome is consistent with the existing literature, which reports the non‐toxic nature of morin hydrate at both cellular and systemic levels in mammals (Rajput et al., 2021). Additionally, this supports the role of this compound in the regulation of sugar metabolism (Hua et al., 2024). Based on these observations, it was confirmed that morin hydrate does not induce toxic effects in mice but has proven to be highly effective in controlling mosquitoes. This suggests a specific mechanism of action against mosquitoes and is responsible for its effects on physiology and metabolism.

## CONCLUSION

Our data indicate that morin hydrate influences the survival, development, fertility and fitness of *A. aegypti* in a dose‐dependent manner. Moreover, there is evidence of residual effects after treatment involving later phases, although further studies are needed to understand the mechanisms of action responsible for these effects. Our analyses revealed that lipid stores were mobilized, and the metabolism of larvae after 24 h of treatment was highly catabolic, even at less lethal concentrations. Additionally, energy metabolism may have been affected at the hormonal level, and energy mobilization is necessary to deal with the impacts of morin treatment. Furthermore, morin hydrate did not exhibit toxicity toward non‐target organisms. However, we reaffirm that there are possible biotechnological applications for the control of *A. aegypti*.

## AUTHOR CONTRIBUTIONS

**Luan Valim dos Santos:** Conceptualization; investigation; writing – original draft; methodology; validation; formal analysis; data curation. **Elaine Rodrigues Miranda Nery da Silva:** Conceptualization; investigation; writing – original draft; methodology. **Matheus Silva Caiado:** Methodology. **Renan Albuquerque Camasmie:** Methodology. **Herbert Marcusi de Souza Agustini:** Methodology. **Raquel do Nascimento de Souza:** Writing – original draft; methodology; formal analysis; data curation. **Bruno Guimarães Marinho:** Methodology; formal analysis; data curation; writing – original draft. **Rosane Nora Castro:** Funding acquisition; writing – review and editing. **Mario Geraldo de Carvalho:** Funding acquisition; writing – review and editing; supervision; resources. **Emerson Guedes Pontes:** Conceptualization; funding acquisition; writing – original draft; writing – review and editing; methodology; formal analysis; data curation; supervision; resources.

## FUNDING INFORMATION

This study was financed in part by the Brazilian agencies Coordenação de Aperfeiçoamento de Pessoal de Nível Superior (CAPES), Finance Code 001; Instituto Nacional de Ciência e Tecnologia em Entomologia Molecular (INCT‐EM); Fundação de Amparo à Pesquisa do Estado do Rio de Janeiro (FAPERJ); and Conselho Nacional de Desenvolvimento Científico e TecnológicoPesquisa (CNPq).

## CONFLICT OF INTEREST STATEMENT

All authors declare that they have no competing interests.

## Supporting information


**Table S1.** Sequences, concentrations and information obtained from calibration curves of primers used in qPCR.

## Data Availability

The data related to the manuscript is available in https://doi.org/10.5061/dryad.x0k6djhwh.
