# Supplementary material for: Morin hydrate reduces survival and fertility, delays development and weakens lipid reserves in Aedes aegypti
Source: Med Vet Entomol. 2025 Apr 19;39(3):592–602. doi: 10.1111/mve.12805 (PMC12323748; doi:10.1111/mve.12805)
Supplement: Supplementary file 1 — Table S1. Sequences, concentrations and information obtained from calibration curves of primers used in qPCR. [file MVE-39-592-s001.docx]

Table S1: Sequences, concentrations, and information obtained from calibration curves of primers used in qPCR

| Gene (VectorBase) | Sequences | Concentration (nM) | Slope | *r*^2^ | Efficiency (%) |
| --- | --- | --- | --- | --- | --- |
| *Actin1* (AAEL011197) | Forward: 5’-CGTCGTGACATCAAGGAAA-3’ | 600 | -3.329 | 0.9992 | 99.67 |
|  | Reverse: 5’-GAACGATGGCTGGAAGAGAG-3’ |  |  |  |  |
| *α-Tubulin* (AAEL013229) | Forward: 5’-CTGCTTCAAAATGCGTGAAT-3’ | 600 | -3.324 | 0.9983 | 99,93 |
|  | Reverse: 5’-GGTTCCAGATCGACGAAA-3’ |  |  |  |  |
| *TAGL1 (AAEL014553)* | Forward: 5’-CATTGGGCACTTGGATTTCT-3’ | 600 | -3.296 | *0,9901* | 101.08 |
|  | Reverse: 5’-GGGCACTTGGTACGCATACT-3’ |  |  |  |  |
| *Brummer (AAEL011918)* | Forward: 5’-TCTTTCGGGTGGTGAACGAG-3’ | 600 | -3.394 | *0.9909* | 97.08 |
|  | Reverse: 5’-GACGTTCTTGCCGTCGTAGA-3’ |  |  |  |  |
| *HSL (AAEL005706)* | Forward: 5’-ATGGAGGAGGGTTCGTAGCT-3’ | 600 | -3.431 | *0.9997* | 95.65 |
|  | Reverse: 5’-CTTCTAGAGCACGCGGGAAT-3’ |  |  |  |  |
| *FAS1 (AAEL001194)* | Forward: 5’-GAGGTCGTCCGATTGGTTTC-3’ | 600 | -3.352 | *0.991* | 98.76 |
|  | Reverse: 5’-AGGACAACCTTGCCGATGTG-3’ |  |  |  |  |
| *GPAT1 (AAEL007080)* | Forward: 5’-AGGTTCTGAAGGCACGCTAC-3’ | 600 | -3.493 | *0.9873* | 93.31 |
|  | Reverse: 5’-GGACGACGAGGATGGTTTGT-3’ |  |  |  |  |
| *DGAT1 (AAEL001204)* | Forward: 5’-GCCGACGTTGTGCTATGAAC-3’ | 600 | -3.342 | *0.985* | 99.18 |
|  | Reverse: 5’-CATGTTGGAGAACGGGACCA-3’ |  |  |  |  |
| *AKHr* (AAEL011325) | Forward: 5’-GCGGCGAAGTGTTGAATTATAG-3’ | 600 | -3.306 | 0.9996 | 100.65 |
|  | Reverse: 5’-CTTGTGTCCATCGTTGAATTGC-3’ |  |  |  |  |
| *ILr (AAEL002317)* | Forward: 5’-GCGGGCAGAGTAAGATCAAG-3’ | 600 | -3.183 | *0,9934* | 106.15 |
|  | Reverse: 5’-ATGTCCCGCAGTTTATCGTC-3’ |  |  |  |  |
